# Dysregulation of complement at the synapse in P301S mice and human tauopathies

**DOI:** 10.1186/s40478-026-02347-2

**Published:** 2026-06-10

**Authors:** Jacqui Nimmo, Nikoleta Daskoulidou, Sophie Van Pottelberge, Louis De Muynck, B. Paul Morgan

**Affiliations:** 1 UK Dementia Research Institute at Cardiff University, Hadyn Ellis Building, Maindy Road, Cardiff, CF24 4HQ UK; 2 Johnson & Johnson, Turnhoutseweg 30, 2340 Beerse, Belgium

**Keywords:** Complement, Tau, P301S, Synapse

## Abstract

Synapse loss is the best correlate of cognitive decline in neurodegenerative diseases (NDDs). In Alzheimer’s disease complement dysregulation, triggered by amyloid-β accumulation, plays a major role in synapse loss, but its contribution in other NDDs where amyloid is absent, most notably tauopathies, is elusive. Aggregation of tau is a prominent co-pathology in many NDDs and is characteristic of classical tauopathies in which amyloid pathology is lacking. Here we explore the effect of tau accumulation on complement dysregulation and its contribution to neuronal damage and synapse loss in a mouse tauopathy model harbouring the familial P301S tau mutation and post-mortem brain samples from human tauopathies. Complement gene and protein expression were analysed in P301S mice at 2, 4 and 6 M of age. Complement dysregulation was evident from qPCR analysis showing increased classical pathway (C4, C2), and complement receptor (C3ar1, Cd11b, Cd11c) gene expression in P301S mice compared to wildtype (WT). C1q protein levels were markedly increased in brain homogenates from P301S mice compared to WT, accompanied by C1q deposition on tau aggregates. Synapse loss was evident for both excitatory and inhibitory synapses and was accompanied by an increased percentage of C1q positive excitatory synapses, unaffected by proximity to tau aggregates. The classic pathway regulator CSMD1 was present on synapses and decreased on C1q positive synapses in P301S mice, implying a loss of protection from complement attack. Observations in human tauopathy brains demonstrated decreased CSMD1-labelled excitatory synapses, consistent with findings in P301S mice. These findings demonstrate that complement dysregulation occurs in areas of tau pathology and may contribute to synapse loss in tauopathies.

**Supplementary Information:**

The online version contains supplementary material available at 10.1186/s40478-026-02347-2.

## Introduction

Complement plays critical roles in driving synapse loss in Alzheimer’s disease (AD) [1–3]. Numerous studies have demonstrated that knock out (KO) of key complement genes, including *C1q*, *C3*, *C6* and *C7*, in AD mouse models confers protection against synapse loss [3–6]; however, the mechanism of complement dysregulation at the synapse remains poorly understood. In AD, studies on complement dysregulation have mainly focused on amyloid pathology, primarily because complement activation products, including the membrane attack complex (MAC) decorate amyloid plaques in post-mortem AD brain [7–9]. Little is known regarding whether AD co-pathologies, most notably tau, are involved in complement dysregulation, or whether complement dysregulation is a feature of neurodegenerative diseases (NDDs) where amyloid pathology is absent, including the tauopathies. We recently reported complement dysregulation on and adjacent to neurons in post-mortem brains across different tauopathy subtypes [10]; here we further address roles of complement in tauopathies utilising a mouse tauopathy model and focussed analyses of human tauopathy brains.

Numerous mouse models of tauopathy have been generated to address disease mechanisms, including several transgenically expressing the human microtubule-associated protein tau (*MAPT*) gene containing tauopathy-associated mutations [11]. Among these, mice overexpressing human Tau containing the MAPT-P301S mutation found in frontotemporal dementia (specifically FTDP-17) have been widely used [12]. These mice display Tau hyperphosphorylation and intraneuronal neurofibrillary tangles (NFTs) in cortex and amygdala; synapse loss and microgliosis are present by three months (M) and neuronal loss is evident from 6 M [12]. Complement dysregulation has been implicated as a driver of neuroinflammation in P301S mice; expression of complement genes, including *C1qa*, *C4* and *C3*, was increased in astrocytes and microglia isolated from hTau.P301S (referred to hereafter as P301S) mice compared to WT [13], and C1q protein was increased in P301S synaptic fractions compared to WT controls [2]. Knockout of *C1q* in P301S mice ameliorated synaptic loss via engulfment by microglia and astrocytes [14], while *C3* knockout ameliorated neurodegeneration and deficits in electrophysiological synaptic activity [13]. Together, these data suggest that complement dysregulation is triggered by the accumulation of tau aggregates in brain independent of amyloid pathology. Whether complement directly causes synaptic dysfunction and loss is uncertain and the contributions of complement regulators at the synapse remains untested.

To address these important knowledge gaps and further our understanding of how complement dysregulation at the synapse may contribute to synaptic dysfunction and loss in tauopathies, we investigated transcriptional changes in complement genes and complement protein expression in brain and at the synapse in P301S mice between 2 M and 6 M of age, the critical period of tau accumulation in the model [12]. The synaptic findings from the model were then validated in human brain samples from two tauopathy subtypes, cortical basal degeneration (CBD) and globular glial tauopathy (GGT).

## Methods

### Animals

All in vivo experiments were conducted in strict accordance with the guidelines of the Association for Assessment and Accreditation of Laboratory Animal Care International (AAALAC), with the European Communities Council Directive of 24th November 1986 (86/609/EEC) and with protocols approved by the local Institutional Animal and Use Ethical Committee. Mice expressing the 0N4R isoform of human *MAPT* with a P301S mutation were obtained from the MRC Laboratory of Molecular Biology, Swindon (UK) and were backcrossed on a C57bl/6 background at the JAX Labs (Bar Harbour, Main, USA). Mice were single housed in an enriched environment in individually ventilated cages and under 12/12 h light/dark cycles (light at 6:00 AM). At 2, 4 and 6 months (M) of age, eight mice (4 female, 4 male for each age group) were sacrificed by decapitation; one brain hemisphere was homogenised for biochemical assays and the other embedded in OCT (VWR, 361603E) and sectioned on a cryostat (Cryostar NX-50, ThermoFisher) at 14 μm thickness for immunohistochemistry (IHC). Sections were stored at -20 °C until use.

### Human brain tissue

Fresh frozen blocks from prefrontal cortex (PFC) Brodmann area 8/9 (PFC, BA8/9) from corticobasal degeneration (CBD, *n* = 5) and globular glial tauopathy (GGT, *n* = 4) donor brains were obtained from the London Neurodegenerative Disease Brain Bank (Kings College London, UK; ethical approval: 23/WA/0124 [REC 3]) (Table 1). Brain tissue samples from non-demented donors matched for age, area and post-mortem delay (*n* = 5) were obtained from the same source and used as controls. Frozen blocks were embedded in OCT and sectioned on a Cryostat at 14 μm thickness.

**Table 1:** Donor details

ID	Age	Sex	PMI	Disease	Duration /yrs	Severity of pathology	Other pathological observations
A305/17	73	M	35.5	CBD	4	Very extensive hyperphosphorylated tau and tau-4R pathology.	Braak tangle stage II Thal phase 4 Early Alzheimer type changes: BNE stage II, mild amyloid angiopathy
A278/12	70	M	6.5	CBD	No data	Extensive tauopathy. Positive for 4 repeat tau and negative for 3 repeat tau in the cortex.	Parenchymal haemorrhage and infarcts > 10 mm diameter
A164/12	73	F	25	CBD	No data	Extensive tauopathy. Severe deposition of tau in brainstem, dentate nucleus, spinal cord	
A082/11	83	M	58	CBD	5	No data	Limbic stage Lewy body pathology
A069/11	84	F	25.5	CBD	8	Severe tau pathology in multiple brain regions.	Braak tangle stage III CERAD no plaques Braak LB stage 0 Thal phase 0 Alzheimer-type tau pathology (age-related) BNE 3
A260/21	71	M	52	GGT	No data	Severe cerebral atrophy, extensive tau pathology.	Infarct/s > 10 mm in diameter Braak tangle stage IV CERAD sparse neuritic plaques Thal phase 1 Moderate/severe arteriosclerosis in occipital white matter Moderate/severe occipital leptomeningeal CAA Braak LB stage not assessed LATE - Negative Lewy Pathology Consensus Absent
A035/20	78	M		GGT	14		
A036/18	68	M	63	GGT	5	Extensive cortical deposition of tau, extensive white matter tau pathology. Progressive cerebral atrophy predominantly in frontal lobes.	Infarct/s > 10 mm in diameter Braak tangle stage IV CERAD no plaques Thal phase 0 TDP43 pathology accompanying AD
A199/15	73	M	67	GGT	No data	Widespread tau pathology.	Mild arteriolar Aß-CAA Braak tangle stage IV CERAD moderate density of neuritic plaques Braak LB stage 0 Globular glial tauopathy Thal phase 1 Alzheimer’s type changes, BNE stage III-IV, disseminated metastatic small cell carcinoma,
A167/16	73	M	24	GGT	No data	Prominent tauopathy.	FTLD-tau TDP43 pathology accompanying AD
A310/09	84	F	35	Control			Tau Braak stage II consistent with normal ageing
A142/16	83	M	48	Control			Tau Braak stage II consistent with ageing
A051/14	76	F	22	Control			Tau Braak stage 2 consistent with ageing
A127/11	73	M	23	Control			Minimal age-related tau. Normal brain.
A273/12	67	M	26	Control			Tau Braak stage 1, very mild ageing changes

### qPCR

For analysis of gene transcription by qPCR, RNA was extracted from fresh frozen blocks of PFC (~ 10–20 mg tissue per extraction) using the RNeasy Lipid Tissue Mini Kit (Qiagen, 74804) following the manufacturer’s instructions. Briefly, tissue was homogenised in 700 µl Qiazol using an automated Bead Bug microtube homogeniser (Sigma Aldrich) for 1 min, then incubated for 5–12 min at room temperature (RT) before adding 200 µl chloroform and mixing on the Bead Bug homogeniser for 15s. The lysed tissue was incubated for 3–10 min at RT and centrifuged at 7800xg for 15 min, supernatant removed, an equal volume of 70% ethanol added, vortexed, applied to RNeasy mini spin columns and centrifuged at 8000xg for 15–30 s. DNA digest was performed using an RNase-Free DNase kit (Qiagen, 79254) following the manufacturer’s instructions. RNA was eluted from the column with 50 µl of RNAse free water and stored at -80 °C until further use. Purity of RNA was confirmed using the NanoDrop Spectrophotometer (ThermoFisher) with anticipated A^260/230^ values of ~ 2. Purified RNA was converted to cDNA using the High-Capacity RNA-to-cDNA™ Kit (ThermoFisher, 4387406) following manufacturer’s instructions.

qPCRs for a panel of mouse complement genes (Online Resource 1) were run in 384-well plates on a QuantStudio Flex 7 Real-Time PCR system (ThermoFisher); cDNA (1 µl) was added to 9 µl of SyberGreen master mix (5ul SyberGreen, 0.5 µl forward primer 10 µM, 0.5 µl reverse primer 10 µM, 3 µl RNAse-free water). Primers were designed with Primer Blast software (National Library of Medicine) and PCR products were verified by gel electrophoresis. *HPRT* was used as a housekeeping gene in all qPCR assays and all samples were run in duplicate with housekeeping genes on each plate. Relative gene expression for P301S mice was normalised to WT at each age. Differences between groups at each age was analysed by 2-way ANOVA with Sidak corrections for post-hoc multiple comparisons.

### Preparation of brain homogenates

Fresh frozen tissue from one brain hemisphere, the brain stem and spinal cord were separately homogenised in RIPA buffer (Pierce, 89900) supplemented with PhosSTOP protease inhibitor (Roche, 04906845001) using an automated Bead Bug microtube homogeniser (Sigma Aldrich). Homogenates were centrifuged at 1000xg for 10 min at 4 °C in a MicroCL 17R centrifuge (ThermoScientific) and the supernatant harvested and stored frozen at -80 °C until use. Protein concentration in the supernatant was measured using the BCA assay (ThermoScientific, 23235).

### Enzyme linked immunosorbent assay (ELISA)

Nunc MaxiSorp™ flat-bottom 96-well plates (Invitrogen) were used for all ELISAs. For mouse iC3b ELISA, plates were coated with monoclonal antibody (mAb) 2/11 (rat anti-mouse C3b/iC3b; Hycult, HM1065), diluted to 5 µg/ml in carbonate buffer (35 mM NaHCO_3_, 15 mM Na_2_CO_3_, pH 9.6), and incubated overnight at 4 °C. The plate was washed 3x in PBS/0.05% Tween 20 (PBSt) and blocked in 3% BSA (100 µl/well) for 5 h at 25 °C. Tissue homogenates were diluted in 0.3% BSA 5 mM EDTA to final concentrations of 4 mg/ml for total brain and brain stem, and 2 mg/ml for spinal cord, added to wells and incubated overnight at 4 °C. Activated serum was used as a standard, titrated from a starting dilution of 1:500 in 0.3% BSA 5 mM EDTA. Plates were washed 3x in PBSt and incubated in biotinylated rabbit anti-mouse C3 polyclonal Ab (in-house; 0.5 µg/ml; 50 µl/well) in 0.3% BSA 5 mM EDTA for 1 h at 25 °C. After washing (3 x PBSt), plates were incubated with peroxidase-conjugated streptavidin (1:200 in 0.3% BSA 5 mM EDTA; R&D Systems, 890803), 50 µl/well for 40 min at 25 °C. Plates were washed as above, developed with O-phenylenediamine dihydrochloride (SIGMAFAST™ OPD, 1003427029, SigmaAldrich), the reaction stopped after 6 min with 10% H_2_SO_4_ and the absorbance at 492 nm read with a FLUOstar™ Omega Microplate Reader.

For mouse C1q ELISA, plates were coated (50 µg/well) in 5 µg/ml mAb 9H10 (mouse anti-mouse C1q; in house [15]) in carbonate buffer for 1 h at 37 °C, washed and blocked as above, tissue homogenates added at 0.3 mg/ml in 0.3%BSA/EDTA and incubated at 37 °C for 2 h. Purified mouse C1q protein was used as a standard, titrated from 2 µg/ml. The plate was washed and incubated in 2 µg/ml biotinylated mAb 2F6 (mouse anti-mouse C1q; in house) for 1 h at RT (50 µl/well) [15]. After washing, peroxidase-conjugated streptavidin was added (1:200 50 µl/well) and incubated at RT for 1 h before washing and developing with OPD substrate. The reaction was stopped after 8 min with 10% H_2_SO_4_ and the absorbance was measured at 492 nm as above.

For terminal complement complex (TCC) ELISA, plates were coated (50 µg/well) with 2.5 µg/ml mAb 22D1 anti-mouse C6 (in house) in PBS overnight at RT, washed and blocked in 3% BSA for 1 h at 37 °C. The plate was washed and tissue homogenates added at 14 mg/ml for brain, 11 mg/ml for brainstem and 6 mg/ml for spinal cord. The assay measures C5b6 in the TCC; hence, purified C5b6 was used as assay standard, titrated from 150 µg/ml. Plates were incubated at 37 °C for 1.5 h, washed and incubated in 5 µg/ml biotinylated Sky59 anti-C5 mAb (Roche), previously shown to cross-react with mouse C5 [16], for 1 h at 37 °C, washed and then incubated in peroxidase-conjugated streptavidin (1:200 50 µl/well) at RT for 30 min before washing and developing with TMB substrate at 37 °C. The reaction was stopped after 20 min with 10% H_2_SO_4_ and the absorbance was measured at 450 nm as above.

For all ELISAs, dilution factors were first determined by titration of homogenates such that samples fell within the linear portion of the log standard curve.

### Immunohistochemistry

For all IHC staining procedures of human and mouse brain, frozen sections were fixed in 90% methanol at -15 °C for 10 min, washed in 0.01 M PBSt, incubated in 10% normal goat serum (NGS) for 1 h at RT and then incubated at 4 °C overnight in primary antibody as described below in 0.01 M PBS, 0.1% triton100 (TxPBS).

For staining and quantification of tau, brain sections from WT and P301S mice were incubated in fluorescently conjugated rabbit anti-Tau-488 (1:200, ThermoFisher, MA5-45934) overnight. Sections were washed and autofluorescence quenched with 0.1% Sudan black B for 10 min at RT. Sections were washed in PBS and mounted in FluoroSave (Millipore, 345789).

For C1q labelling of neurons in mice, P301S mouse brain Sections (4–6 M, an age when tau is present) were stained with rabbit anti-C1q mAb (1:1000, Abcam 182451) overnight at 4 °C, washed in PBSt and incubated in goat anti-rabbit IgG AlexaFluor-594 (1:500) 1 h at RT. After washing in PBSt, sections were incubated in fluorescently conjugated rabbit anti-Tau-488 (1:200, ThermoFisher, MA5-45934) and rabbit anti-NeuN-405 (1:100, Bio-Techne, NBP3-14002AF405) at 4 °C overnight, washed in PBSt, quenched with Sudan black B and mounted as described above.

For synaptic staining of mouse and human cases, C1q-tagged synapses were detected with rabbit anti-C1q mAb (1:1000, Abcam 182451), co-staining with guinea pig anti-Bassoon (1:1000, Synaptic Systems, 141 318), and chicken anti-Homer1 (1:1000, Synaptic Systems, 160006) for excitatory synapses, or chicken anti-Gephyrin (1:500, Synaptic Systems, 147 009) for inhibitory synapses. Sections were washed in PBSt before incubating in goat anti-rabbit IgG Alexa fluor-488 (Invitrogen, A11034), goat anti-guinea pig IgG Alexa fluor-594 (Invitrogen, A11076), goat anti-chicken IgG Alexa fluor-647 (Invitrogen, A32933), all at 1:500 dilution in TxPBS for 1 h at RT. For analysis of synaptic proximity to tau-positive neurons, the P301S mouse brain sections were further incubated in AlexaFluor 405 conjugated anti-Tau antibody (1:100, Bio-Techne, NBP3-18265AF405, recognising epitope pSer202/Thr205) for 2 h at 37 °C. To identify neurons without tau, a second set of synaptic labelled tissue sections were incubated in rabbit anti-Tau-488 (1:200, ThermoFisher, MA5-45934) and rabbit anti-NeuN-405 (1:100, Bio-Techne, NBP3-14002AF405) at 4 °C overnight. Tau-positive neurons could not be imaged or quantified in this second set due to overlapping fluorophores with C1q. Sections were washed, autofluorescence quenched with 0.1% Sudan black B for 10 min at RT, washed in PBS and mounted in FluoroSave (Millipore, 345789).

For CSMD1 labelling of human and mouse synapses, tissue was first blocked with endogenous biotin blocking kit (ThermoFisher, E21390) following manufacturer’s instructions, then incubated in 10% NGS (1 h RT) followed by primary antibody mix containing rabbit anti-C1q mAb (1:1000, Abcam 182451), guinea pig anti-Bassoon (1:1000, Synaptic Systems, 141 318), and biotinylated mouse anti-CSMD1 (10 µg/ml, mAb 3D10, in house [17]), at 4 °C overnight. Sections were washed in PBSt before incubating in goat anti-rabbit IgG Alexa fluor-647, goat anti-Guinea pig IgG Alexa fluor-594 (both at 1:500 dilution) and streptavidin Alexa fluor-488 (1:1000) in TxPBS for 1 h at RT. Autofluorescence was quenched and sections mounted as previously described.

### Image analysis

All images were captured on a Leica SP8 confocal microscope (ThermoFisher). For analysis of tau load, 6 images from layers 3–5 of the somatomotor and somatosensory cortex were captured over two consecutive brain sections per mouse (20x objective; image size 184.52 µm^2^; 5 μm z-stack with 1 μm step size). Images were analysed in FIJI software to quantify the average percentage area of tau immunostaining in WT and P301S mice at each age.

For C1q labelled neurons, 10 images from layers 3–5 of the somatomotor and somatosensory cortex were captured over two consecutive brain sections per mouse (40x objective; image size 184.52 µm^2^; Single plane). Images were imported into Imaris for quantification of the mean fluorescence intensity of C1q within each NeuN-positive or tau-positive neuron and average values were plotted per mouse.

For synaptic analysis, eight images from layers 3–5 of the somatomotor and somatosensory cortex were captured over two consecutive brain sections per mouse (63x objective; image size 184.52 µm^2^; Single plane). Images were then cropped in Leica software into 30 µm^2^ regions for synapse analysis. Crops were imported into Imaris (version 10.0.0, Bitplane, Zurich, Switzerland) and batch processed for quantification of synapses using spot analysis with diameter 0.4 μm. Viable synapses were quantified when pre- and post-synaptic markers were co-localised within 0.37 μm of each other. Synapses were classified as excitatory when co-localised with Bassoon (universal presynaptic marker) and Homer1 positive spots, or inhibitory when co-localised with Bassoon and Gephyrin positive spots. The percentages of C1q or CSMD1 positive synapses were quantified from co-localisation of C1q or CSMD1 spots within 0.37 μm of bassoon positive spots.

For analysis of synaptic C1q labelling relationship to proximity to neurons, images were cropped into 50 µm^2^ regions. Synapses were classified in Imaris as described and the percentage of C1q positive synapses (colocalised within 0.37 μm distance from Bassoon and Homer1) quantified within concentric zones at 0–2, 2–7, 7–15 and > 15 μm from the neuronal soma.

### Statistics

In all data sets, there was no difference between male and female mice; genders were therefore combined for statistical analysis. Statistical analysis was performed on average values in GraphPad Prism V10 software. All bar graphs represent mean values with each data point representing an individual mouse in each study. One-way ANOVA, two-way ANOVA or t-tests were performed as appropriate and stated in the text.

## Results

### Transcriptional changes in complement genes with age and pathology in P301S mice

**Fig. 1:**
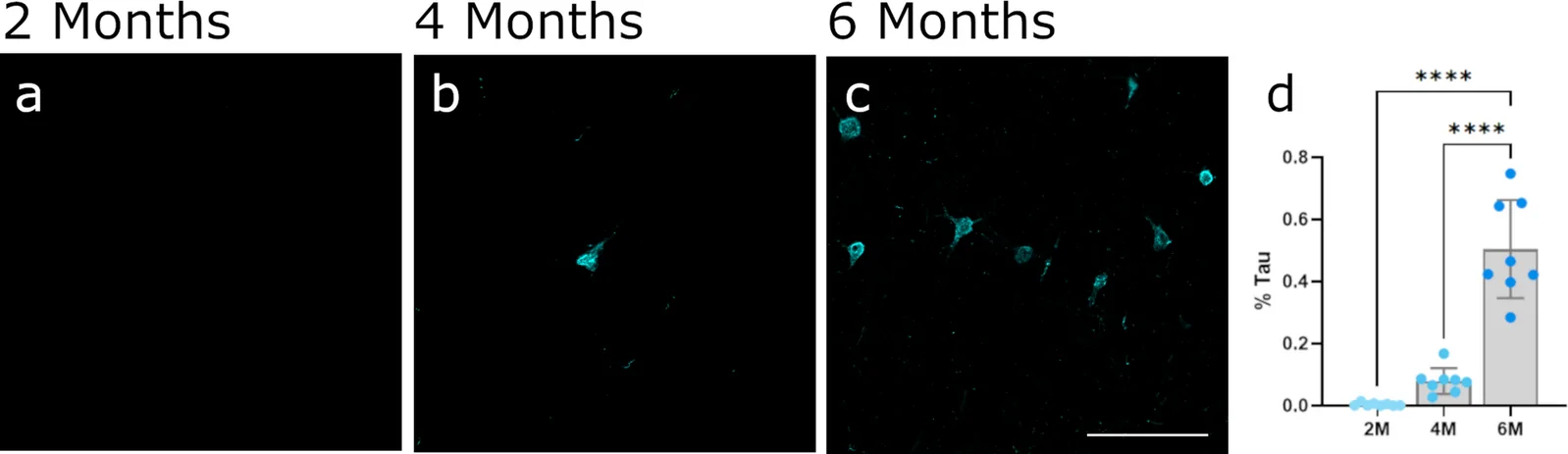
Progression of tau pathology in P301S mice. Immunolabelling of hyperphosphorylated tau (MA5-45934, Cyan) at 2 M (**a**), 4 M (**b**) and 6 M (**c**), *n* = 8 per group (4 male, 4 female). Quantification of the tau immunolabelling in each mouse demonstrates significant increase in % tau at 4 and 6 M compared to 2 M when there is no detectable tau (**d**). Differences between groups were compared by one-way ANOVA with Tukey’s corrections for post-hoc comparisons (F [2, 21] = 65.38, *p* < 0.0001). Error bars represent mean ± SD; ****p < 0.0001. Scale bar represents all images = 50 μm

In P301S mice, tau pathology was absent at 2 M, present at a low level in all mice at 4 M and abundant in all mice at 6 M, highly significantly increased compared to earlier ages (Fig. 1); as expected, tau pathology was absent in WT mice at all ages.

**Fig. 2:**
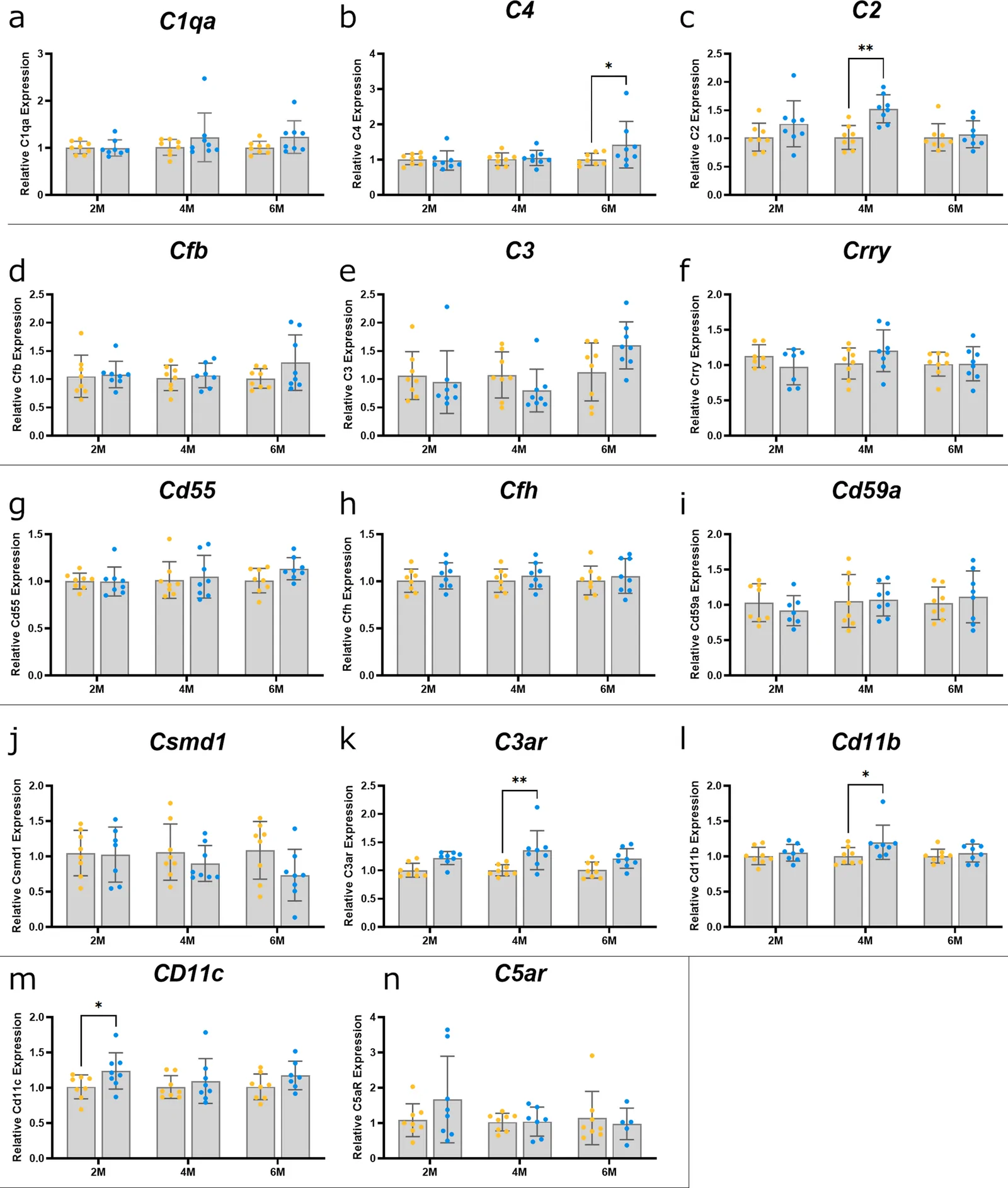
Expression of complement genes. Complement gene expression was compared using qPCR in prefrontal cortex from P301S (blue) and WT (yellow) mice at 2, 4 and 6 M, (*n* = 4 female and *n* = 4 male for each age group). Expression of genes encoding complement components *C1qa* (**a**), *C4* (**b**), *C2* (**c**), *Cfb* (**d**) and *C3* (**e**), regulators *Crry* (**f**), *Cd55* (**g**) *Cfh* (**h**), *Cd59* (**i**) and *Csmd1* (**j**), and receptors *C3ar1* (**k**), *Cd11b* (**l**) and *Cd11c* (**m**) were compared between groups in P301S and WT by two-way ANOVA with Sidak’s corrections for post-hoc analysis. Significant increases in relative gene expression was observed for *C4* (F[1,42] = 2.18, *p* = 0.1472; WT-P301S 6 M *p* = 0.044) (**b**), *C2* (F[1,42] = 23.34, *p* < 0.0001; WT-P301S 4 M *p* = 0.018) (c) *C3aR* (F[1,42] = 2.18, *p* = 0.1472; WT-P301S 4 M *p* = 0.0012) (**k**) *Cd11b* (F[1,42] = 4.9, *p* = 0.0309; WT-P301S 6 M *p* = 0.0325) (l) and *Cd11c* (F[1,41] = 5.86, *p* = 0.0200; WT-P301S 2 M *p* = 0.0495). Error bars represent mean ± SD; *p = 0.05− 0.01, **p = 0.01–0.005

RNA was extracted from the prefrontal cortex of WT and P301S mice at 2, 4 and 6 M of age (*n* = 4 males and *n* = 4 females for each line at each age). Transcriptional changes were assessed by qPCR for genes encoding the central complement component C3 (*C3*), classical (*C1qa*,* C4*,* C2*) and alternative (*Cfb*) pathway components, complement regulators (*Cfh*,* Csmd1*, *Cd55*,* Crry*,* Cd59a*) and receptors (*Cd11b*,* Cd11c*,* C3ar1*,* C5ar1*); significance of the impact of genotype on transcriptional changes was assessed by 2-way ANOVA (Fig. 2). Expression of terminal pathway genes encoding components of the membrane attack complex (*C5-C9*) were below detection threshold and were omitted from the panel [18].

For classical pathway genes, there was no significant difference in *C1qa* expression between P301S and WT at any age (Fig. 2a); however *C2* expression was significantly increased in P301S mice compared to WT at 4 M (Fig. 2c). There was no difference in expression of *C3* or *Cfb* between P301S and WT mice at any age (Fig. 2d, e). Expression of genes encoding complement regulators *Crry* (Fig. 2f), *Cd55* (Fig. 2g), *Cfh* (Fig. 2h), *Cd59a* (Fig. 2i) or *Csmd1* (Fig. 2j) were not different between P301S and WT mice at any age. Of the genes encoding complement receptors, no difference was observed in *C5ar1* expression; however, significantly higher expression levels were observed in P301S compared to WT for *C3ar1* at 4M, *Cd11b* at 4 M and *Cd11c* at 2 M (Fig. 2k–l).

### Complement protein changes with age and pathology in P301S brain

**Fig. 3:**
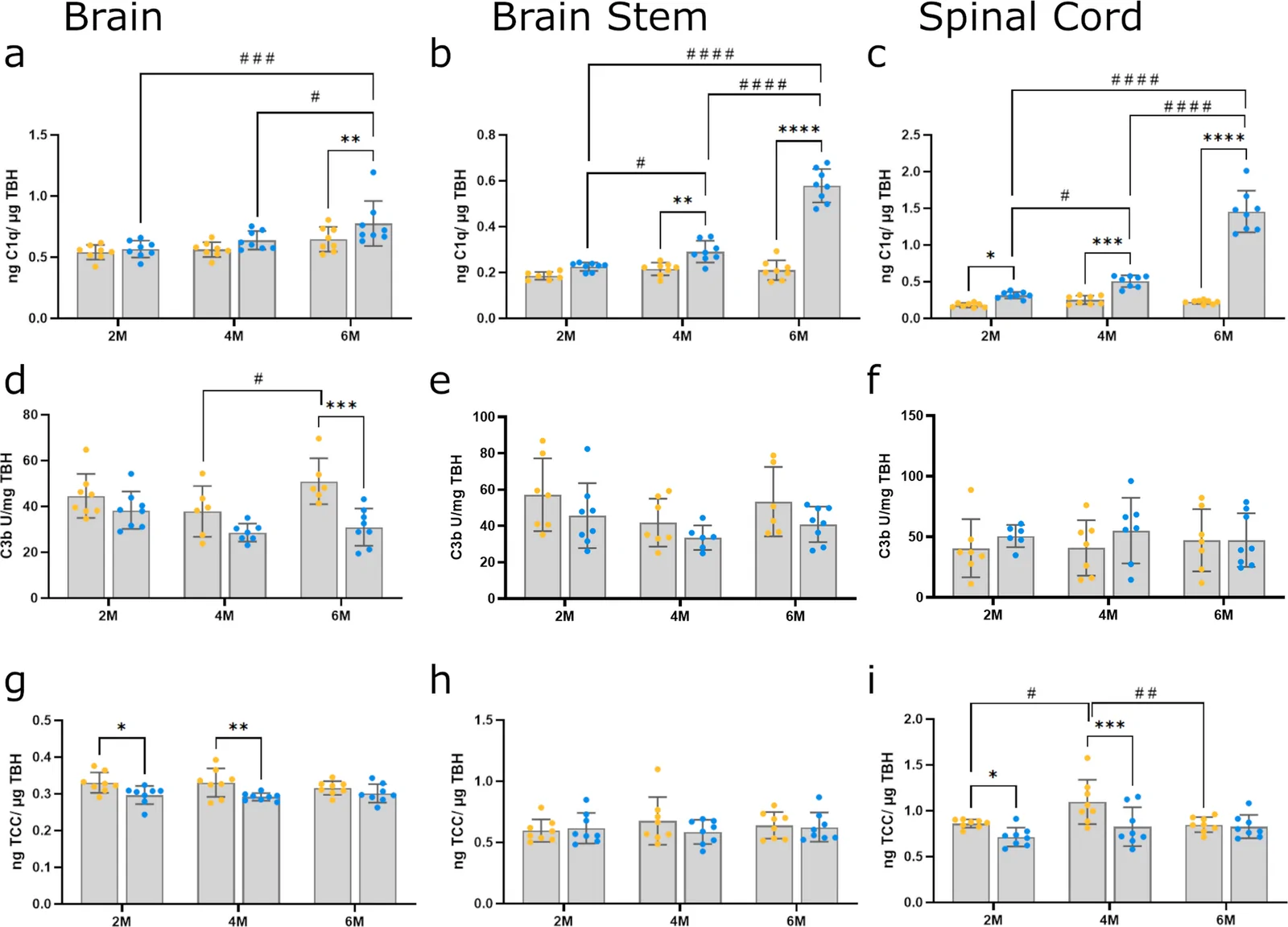
C1q, C3b and TCC ELISA in brain homogenates. C1q (**a**–**c**), C3b (**d**–**f**) and TCC (**g**–**i**) were measured in brain (**a**, **d**, **g**), brain stem (**b**, **e**, **h**) and spinal cord (**c**, **f**, **i**) from WT (yellow) and P301S (blue) mice at 2, 4 and 6 M of age (*n* = 4 female and *n* = 4 male for each age group). Differences between groups were assessed for significance by two-way ANOVA with Sidak’s corrections for post-hoc analysis. Two-way ANOVA showed significant effect of age and genotype for C1q levels in brain (Genotype: F [2, 21] = 8.592, *p* = 0.0019; Age: F [1, 21] = 8.554, *p* = 0.0081), brain stem (Genotype: F[1,41] = 166.3, *p* < 0.0001; Age: F[2,41] = 82.21, *p* < 0.0001) and spinal cord (Genotype: F [1, 21] = 215.2, *p* < 0.0001; Age: F [2, 21] = 103.1, *p* < 0.0001). Two-way ANOVA showed significant effect of age and genotype for C3b/iC3b levels in brain (Genotype: F[1,37] = 19.63, *p* < 0.0001; Age: F[2,37] = 3.864, *p* < 0.0001). Two-way ANOVA showed significant effect of age for TCC levels in brain (Age: F [1, 21] = 16.48, *p* = 0.0006) and age and genotype for spinal cord (Genotype: F [1, 21] = 14.58, *p* = 0.001; Age: F [2, 21] = 4.461, *p* = 0.0243). *p = 0.05−0.01, **p = 0.01 − 0.005, ***p = 0.005−0.001. Error bars represent mean ± SD

To further investigate the extent of complement activation, protein expression levels of activation pathway (C1q; C3b/iC3b) and terminal (TCC) pathway components were measured by ELISA in total brain homogenates. Tau pathology in P301S mice is widespread, more prominent in spinal cord and brainstem regions [12, 19]; we therefore analysed complement protein levels separately in brain, brainstem and spinal cord. Levels of C1q, C3b/iC3b and TCC were expressed relative to total protein. C1q levels in each central nervous system (CNS) region increased with age in P301S but not WT mice, significantly higher in P301S at 6 M compared to either 2 or 4 M (Fig. 3a–c). Comparing P301S to WT mice, C1q levels were significantly higher in the spinal cord of P301S mice at each age, 7-fold higher at 6 M (Fig. 3c); in brainstem C1q levels were significantly higher in P301S mice at 4 M and 6 M, 3-fold higher at 6 M (Fig. 3b), while in brain C1q levels were only significantly increased at 6 M (Fig. 3a). In general, C3b/iC3b levels were unchanged across genotype and age with little variation in brainstem and spinal cord (Fig. 3d–f); however, in brain C3b/iC3b was significantly reduced in P301S compared to WT at 6 M (Fig. 3d).

TCC levels in brain and spinal cord trended lower in P301S compared to WT, significant at 2 M and 4 M, (Fig. 3g, i); in brainstem, TCC levels were not different between the lines or ages.

### C1q is deposited on neurons with tau aggregates

**Fig. 4:**
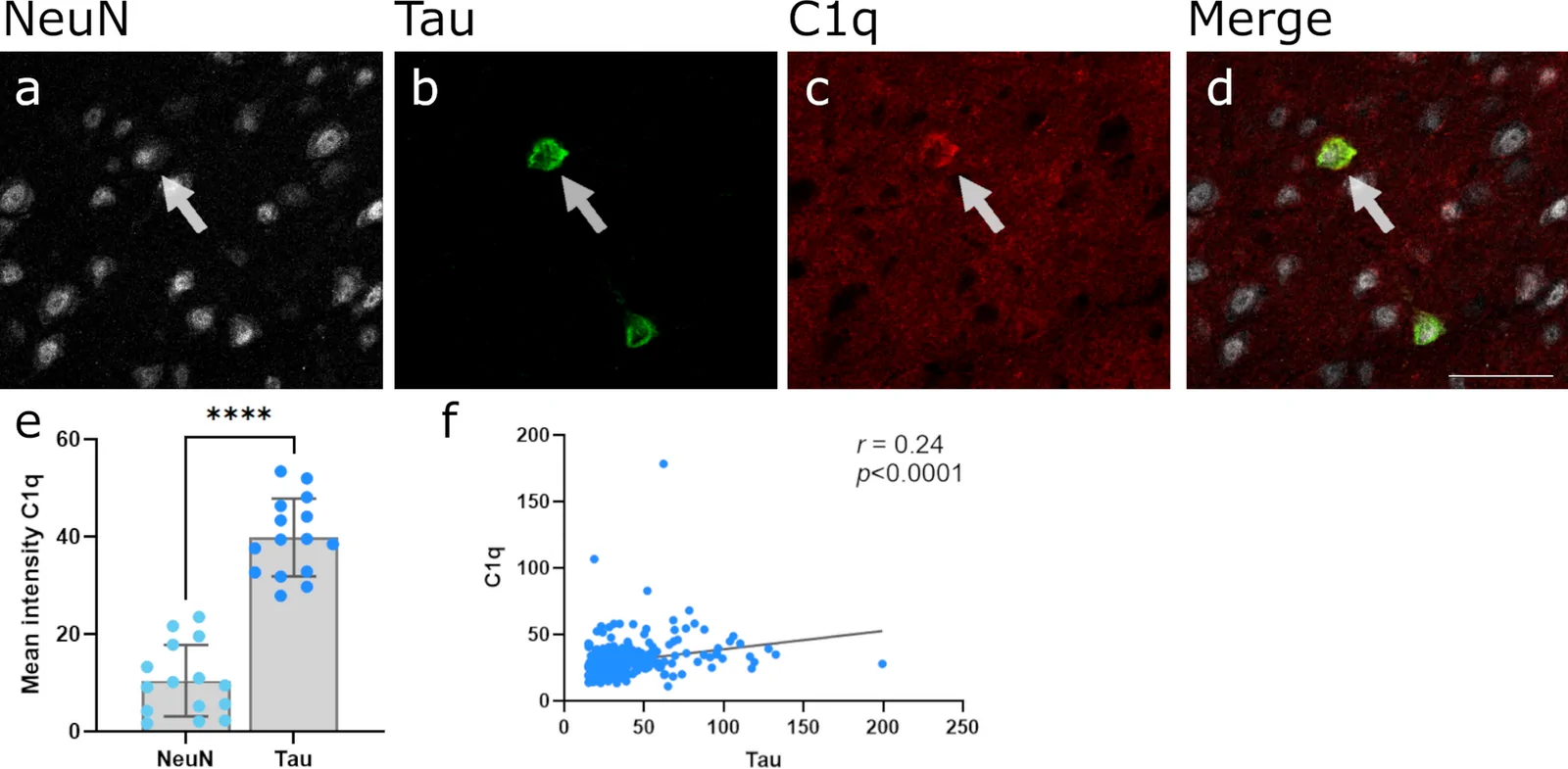
C1q deposition on neurons in P301S mice. a-d Representative IHC images of the neuronal marker NeuN (**a**; white), **b** hyperphosphorylated tau (**b**; green), and C1q (**c**; red); merged in **d**. C1q is largely deposited on tau-positive neurons (arrow). Quantification of the mean fluorescent intensity of C1q on neurons confirmed the highly significant deposition of C1q on Tau-positive neurons compared to Tau-negative NeuN-positive neurons (**e**), (P301S mice 4–6 M, *n* = 16). Pearson correlation analysis of the mean fluorescent intensity of tau and C1q on neurons showed significant positive correlation (*r* = 0.24, *p* < 0.0001). Error bars represent mean ± SD; ****p < 0.0001. Scale bar in d represents all images = 50 μm

C1q deposition on neurons with and without tau aggregates was assessed in cortical layers 3–5 of P301S mice at 4 and 6 M (Fig. 4), ages where tau is evident (Fig. 1). Neurons with aggregated tau (positive for both tau and NeuN) had markedly higher intensity of C1q staining; indeed, neurons without tau aggregates (NeuN-positive and tau-negative) expressed little C1q (mean fluorescent intensity: tau-positive neurons 39.8, tau-negative 10.4; *p* < 0.0001; Fig. 4a–e). A correlation analysis comparing neuronal levels of aggregated tau and C1q staining by Pearson analysis showed a significant positive correlation (*r* = 0.31, *p* < 0.0001; Fig. 4f).

### Loss of excitatory and inhibitory synapses with tau tangle pathology in P301S mice

**Fig. 5:**
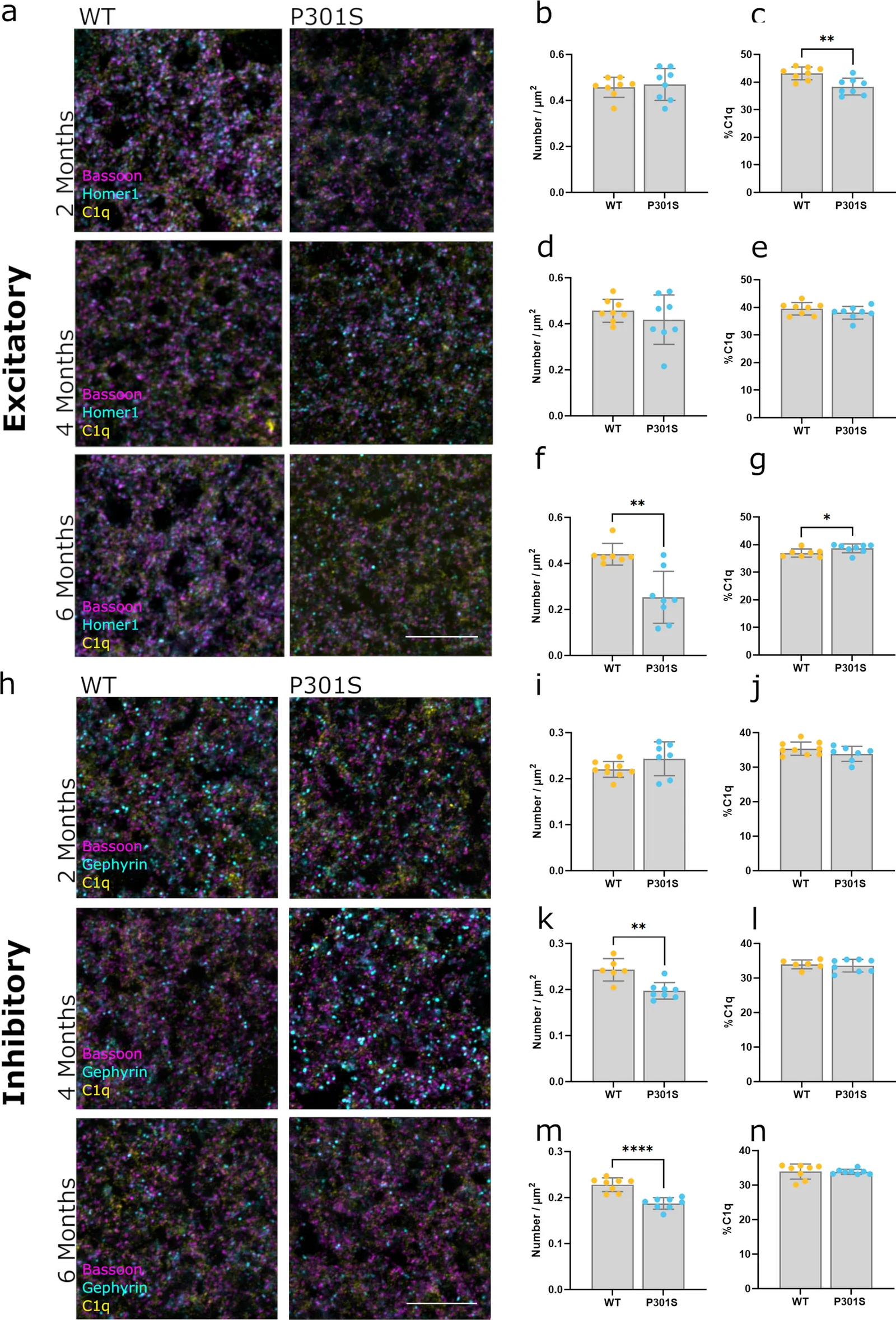
C1q labelling of excitatory and inhibitory synapses. C1q labelling (yellow) of excitatory (colocalization of pre-synaptic marker bassoon (magenta) and post-synaptic marker Homer1 (cyan); **a**–**g** and inhibitory (colocalization of bassoon (magenta) with post-synaptic marker Gephyrin (cyan); **h**–**n** synapses was quantified in WT and P301S mice at ages 2 M (**b**, **c**, **i**, **j**), 4 M (**d**, **e**, **k**, **l**) and 6 M (**f**, **g**, **m**, **n**), *n* = 8 per group (4 female, 4 male). Synapse density was significantly decreased for excitatory and inhibitory synapses in P301S compared to WT at 4 M (**k**) and 6 M (**f**, **m**). The percentage of C1q-labelled excitatory synapses but not inhibitory synapses was significantly reduced at 2 M (**c**, **g**) and increased at 6 M (**g**, **n**). Differences between groups was assessed by two tailed *t* test. Error bars represent mean ± SD; *p < 0.05, **p < 0.01, ***p < 0.001, ****p < 0.0001. Scale bars represent all images = 10 μm

Synaptic density was assessed by immunohistochemistry at different ages of P301S and WT mice. Tissue was co-labelled for synapses and tau and images captured within cortical layers 3–5 where tau aggregates are most prominent. The subset of viable synapses was identified by co-localisation (inter-spot distance of < 0.37 μm) of the pre-synaptic marker Bassoon with postsynaptic markers Homer1 for excitatory (Fig. 5a–g) or Gephyrin for inhibitory (Fig. 5h–n) synapses respectively. Excitatory synapses were significantly reduced (by ~ 50%; *p* < 0.01) in P301S compared to WT mice at 6 M (Fig. 5f). Inhibitory synapses were also significantly decreased in P301S compared to WT mice at 4 M and 6 M (by ~ 20%; *p* < 0.001; Fig. 5k, m). In both cases this was due to loss of both pre and post synaptic terminals (Online Resource 2).

Co-labelling of synapses for C1q showed that the proportion of excitatory synapses positive for C1q was significantly lower at 2 M in P301S mice compared to WT (Fig. 5c), but at 6 M the proportion of C1q-positive excitatory synapses showed a small but significant increase in P301S mice compared to WT, indicating that C1q may be labelling synapses for phagocytosis during this period of active synapse loss (Fig. 5g). In contrast, no difference in the percentage of C1q-labelled inhibitory synapses was observed at any age (Fig. 5j, l, n); this may reflect the observed smaller reduction in inhibitory compared to excitatory synapses in P301S mice.

**Fig. 6:**
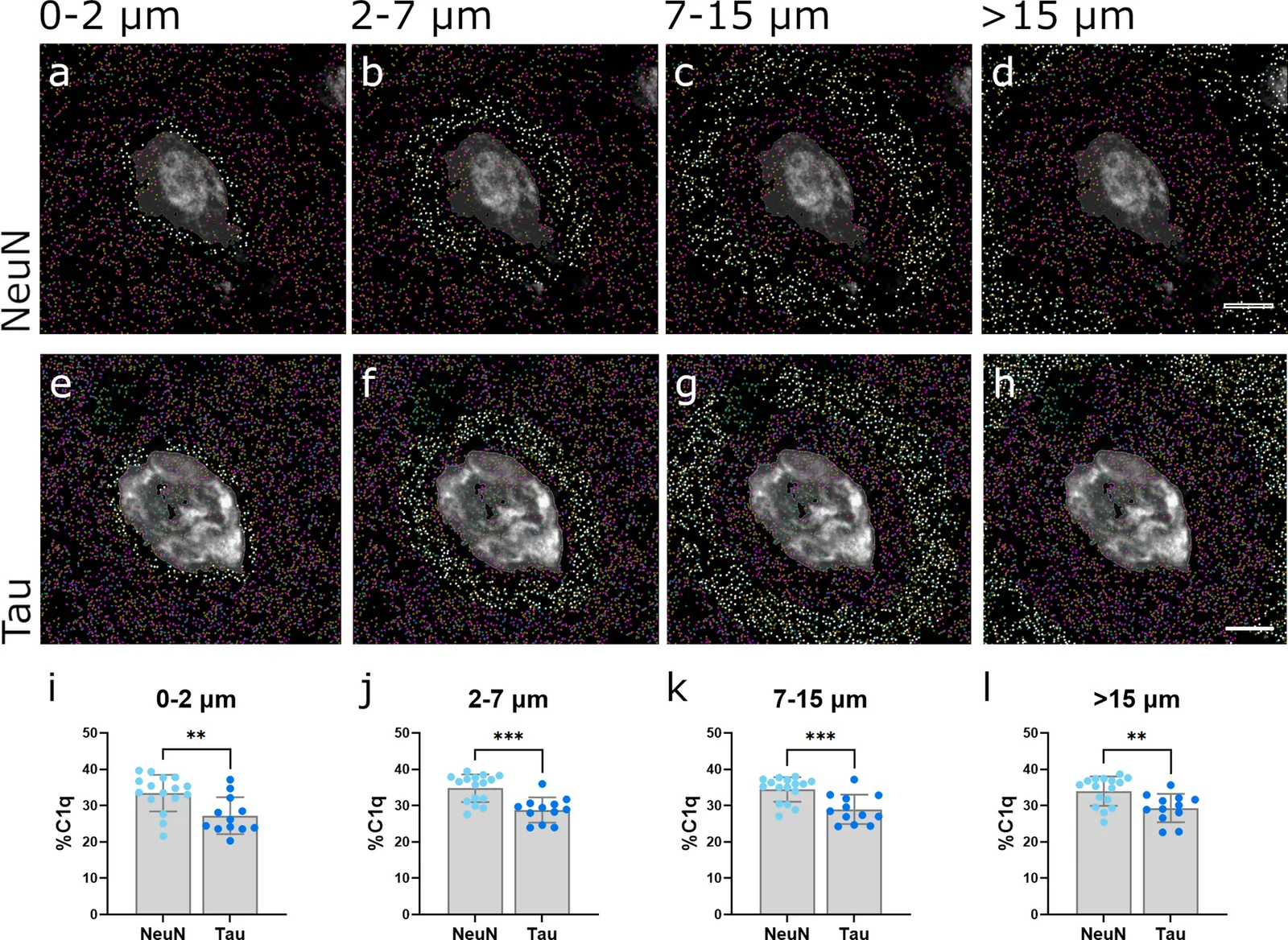
Impact of tau pathology on C1q labelling of excitatory synapses. The percentage of excitatory synapses (stained for Bassoon (magenta) and Homer1 (Cyan)) labelled with C1q (yellow) was analysed in proximity to healthy neurons (NeuN, white) or intraneuronal tau aggregates (Tau, White) in 4-6 M P301S mice (*n* = 12–16). Excitatory synapses were isolated and quantified in Imaris within zones of increasing distance from neuronal soma at 0–2 μm (**a**–**e**), 2–7 μm (**b**–**f**) 7–15 μm (**c**–**g**) and > 15 μm (**d**–**h**); Images represent processed images from Imaris with zones highlighted in white. Differences between groups was assessed by one-way ANOVA with Dunnett’s corrections for post-hoc analysis. Quantification of %C1q labelled synapses showed a significant decrease in zones surrounding tau positive neurons compared to healthy neurons (**i**–**l**) (*n* = 16, 4–6 M). Error bars represent mean ± SD. *p = 0.05. Scale bar = 8 μm

To investigate whether C1q tagging of excitatory synapses was influenced by proximity to neurons with or without tau tangles, brain sections were labelled for C1q, excitatory synaptic markers (Bassoon, Homer1) and either tau to identify tangles, or tau and NeuN to identify neurons that did not have tau aggregates (Fig. 6). When assessed in zones within a 0–2 μm, 2–7 μm, 7–15 μm and > 15 μm radius around the neuronal soma, analysed in Imaris (Fig. 6, NeuN: a–d, tau: e–h), the percentage of C1q-positive synapses was ~ 20–30% lower around tau-positive neurons compared to tau-negative (NeuN-stained) neurons (Fig. 6i–l). This may reflect the loss of C1q-labelled synapses around tau positive neurons.

### Number of CSMD1 and C1q positive synapses is decreased in P301S mice

**Fig. 7:**
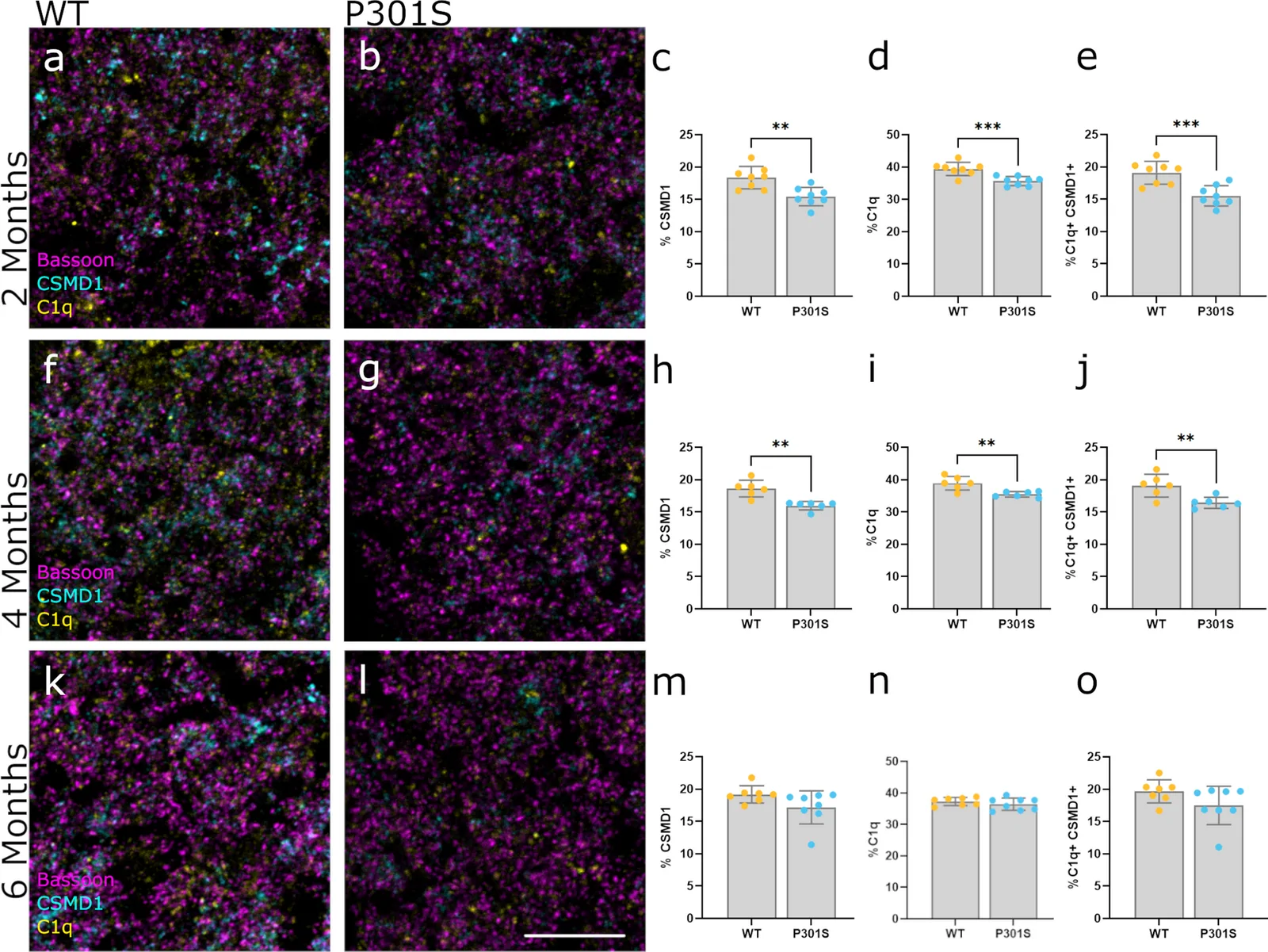
CSMD1 labelling of synapses. CSMD1-positive, C1q positive and CSMD1/C1q double positive synapses were quantified from colocalization of the presynaptic marker Bassoon (Magenta) with CSMD1 (Cyan) and/or C1q (Yellow) in WT and P301S mice at 2 M (**a**–**e**), 4 M (**f**–**j**) and 6 M (**k**–**o**) of age (*n* = 4 female and *n* = 4 male for each age group). The percentage of CSMD1 + synapses (**c**, **h**, **m**), C1q+ synapses (**d**, **i**, **n**) and C1q+CSMD1 + synapses (**e**, **j**, **o**) were significantly reduced in P301S compared to WT mice at 2–4 M of age. Differences between groups was assessed by two tailed t-test. Error bars represent mean ± SD; *p < 0.05, **p < 0.01. Scale bar represents all images = 10 μm

We and others have recently demonstrated that the classical pathway (CP) complement regulator CSMD1 is located on synapses in both mice and man, implicating CSMD1 in synapse survival [17, 20, 21]. To investigate the balance between CP complement activation and regulation at the synapse, immunolabelling of C1q (a marker of CP activation) and CSMD1 (a regulator of the CP) at Bassoon-positive synapses was performed. The percentages of synapses positive for C1q, positive for CSMD1 and double-labelled for C1q and CSMD1 were quantified in cortical layers 3–5 where tau aggregates are most prominent in P301S and WT mice (Fig. 7). Tissue was co-labelled for synapses and tau and images captured; the spectral overlap of available AlexaFluor secondary antibodies limited simultaneous detection to four antibody markers; hence, we chose to measure total Bassoon-positive synapses, not excitatory or inhibitory synapses, explaining the difference in percentage of C1q positive synapses compared to excitatory synapses alone in P301S mice at 6 M (Fig. 5c). In this analysis, the percentage of all synapses that were positive for C1q was significantly lower in P301S mice compared to WT at 2 M and 4 M (Fig. 7d, i), whereas no difference was observed at 6 M (Fig. 7n). Both the percentage of total CSMD1 positive synapses (Fig. 7c, h) and the percentage of C1q labelled synapses that were also positive for CSMD1 (Fig. 7e, j) was significantly decreased at 2 M and 4 M in P301S mice compared to WT, no difference was observed at 6 M (Fig. 7m–o). These data suggest that loss of the CP regulator CSMD1 increases synapse vulnerability to complement attack.

### CSMD1 positive synapses are decreased in human tauopathies

**Fig. 8:**
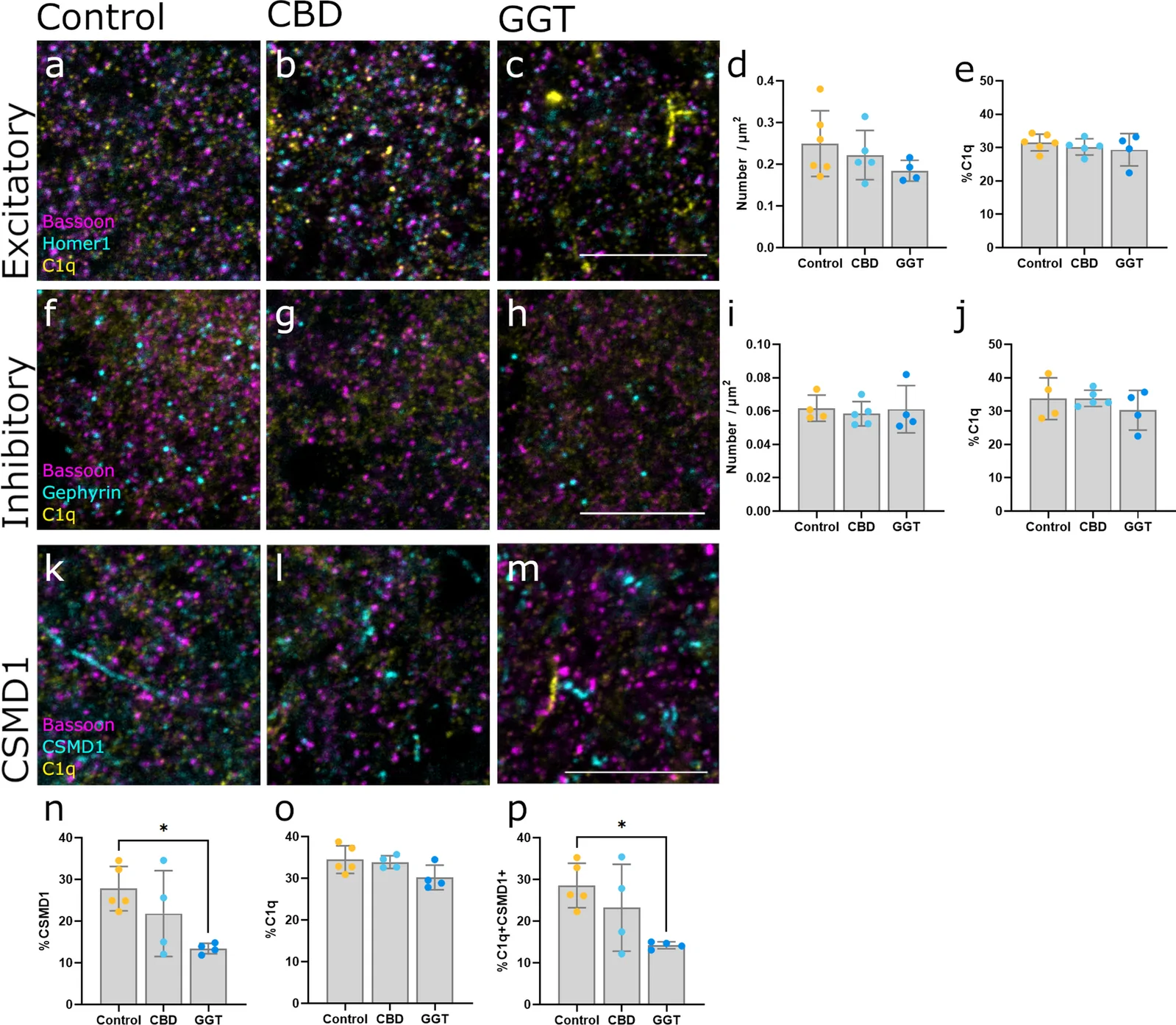
**Synaptic C1q and CSMD1 staining in human tauopathy cases. **Brain sections from corticobasal degeneration (CBD, *n* = 5) and Globular glial tauopathy (GGT, *n* = 4) cases were compared to healthy control (*n* = 5). Synapses were labelled with the presynaptic marker bassoon (Magenta); excitatory and inhibitory synapses were distinguished by the postsynaptic markers Homer1 (Cyan, **a**–**e**) and Gephyrin (Cyan, **f**–**j**) respectively. Synapses were additionally stained for C1q (Yellow) and CSMD1 (Cyan, **k–m**). Synaptic density and percentage of C1q+ synapses was quantified for both excitatory (**d**–**e**) and inhibitory (**i**–**j**) synaptic populations with no significant difference seen between tauopathy and control groups. The percentage of synapses staining for CSMD1 and double-stained for CSMD1 and C1q were assessed only in total bassoon positive synapses because of detection availability (**n**–**p**). There was a significant decrease in the percentage of CSMD1+ (**n**) and C1q+CSMD1+ (**p**) synapses in GGT compared to controls; Differences between groups was assessed by two tailed t-test. Error bars represent mean ± SD. *p < 0.05. Scale bar represents all images = 10 μm

To test whether a similar pattern of synapse loss occurred in human tauopathies, tissue from two primary tauopathy disease subtypes, CBD (5 cases) and GGT (4 cases), were selected and compared to healthy controls (6 donors) (Fig. 8). Staining and synaptic classification was conducted as for mouse brain tissue. In contrast to mouse brain, there was no significant difference in synaptic density; however, a non-significant trend towards decreased excitatory synapses was observed in GGT compared to control cases (Fig. 8d). As in the mouse model, 30–40% of excitatory or inhibitory synapses were colocalised with C1q; however, there was no difference between disease and control cases (Fig. 8e, j).

Synaptic expression of the classical pathway regulator CSMD1 and co-expression of C1q and CSMD1 was also analysed on total Bassoon-positive synapses in the human cases (Fig. 8k–p). Consistent with results in P301S mice, in GGT cases there was a significant reduction in the percentage of total CSMD1 labelled synapses (Fig. 8n) compared to healthy controls as well as the percentage of C1q positive synapses that were also labelled with CSMD1 (Fig. 8p).

## Discussion

Dysregulation of the complement system has been implicated as a driver of neurodegeneration and synaptic loss in AD and mouse models of AD where amyloid deposition is considered the primary initiator of complement activation [4]. The role of tau as a driver of complement dysregulation has been overlooked in most of these studies despite evidence that tau accumulation, together with synaptic loss, is a major correlate of cognitive decline in dementia [22]. Our recent work demonstrates that complement activation products are increased in post-mortem brain tissue from tauopathy subtypes at advanced stages of disease [10]. This is consistent with a few studies in rodent tauopathy models, notably P301S mice, which show that tau, independent of amyloid, can activate complement and play a role in synapse loss [14, 23]. However, mechanisms of complement dysregulation at the synapse and the contribution of complement regulators has not been established. To address this, we explored complement expression and its association with disease hallmarks in P301S mice and post-mortem human brain tissue from two tauopathy types, CBD and GGT. The P301S mouse line used here expresses the human 0N4R tau isoform harbouring the familial P301S mutation under the Thy1 promoter to induce neuronal expression of tau [12]. Hyperphosphorylated tau is evident in the brain from 4 M with significant tau pathology observed by 6 M of age (Fig. 1) [12]. In our study, P301S mice were used at 2, 4 and 6 M, enabling complement dysregulation to be explored with increased age and progression of tau pathology.

We demonstrated early transcriptional upregulation in P301S mice of genes encoding C4 and C2, key components involved in formation of the amplifying C3 convertase of the CP (C4b2a), along with the iC3b-binding phagocytic receptor CR3, and the pro-inflammatory receptor C3aR1 which binds C3a (Fig. 2k-m). CR3 is known to be expressed on microglia [3]; here we observed that upregulation of CR3 occurred at 4 M when significant synapse loss is observed in P301S mice, suggesting that increased expression of CR3 on microglia may facilitate phagocytic elimination of synapses.

Notably, C3aR1 was significantly upregulated at 4 M in P301S mice compared to WT controls (Fig. 2k). Upregulation of C3aR1, predominantly expressed on microglia [3, 24], implies a contribution to neuroinflammation and pathology in the model. Although the observation that levels of the C3 activation products (C3b/iC3b) and TCC were not significantly different between controls and P301S mice implies minimal complement activation in the model, this may not be representative of the total C3 and TCC activation as these may be locked to targets in the tissue, rendering them difficult to extract and measure in brain homogenates. The C3a activation product which binds to the C3aR is soluble and hence not locked to targets; however, C3a was not measured by ELISA in this study.

Increased expression of genes encoding the CP components C2 and C4 was evident at 4 and 6 M, after the onset of tau accumulation, suggesting CP dysregulation at these stages (Fig. 2b, c). Although genes encoding C1q were not significantly upregulated at the transcriptional level, C1q protein levels were markedly and significantly elevated in brain homogenates from P301S mice at 6 M (Fig. 3a) reflecting the marked increase in the percentage of C1q labelled excitatory synapses and synaptic loss at this age (Fig. 5f, g). C1q levels in homogenates of P301S brainstem and spinal cord, regions known to have more severe tau pathology and neuronal degeneration compared to brain, showed markedly increased C1q levels compared to WT, not only at 6 M (7-fold increased in spinal cord) but also at 2 M in spinal cord (Fig. 3c) and 4 M in brainstem and spinal cord (Fig. 3b, c). The low levels of C1q in WT CNS regions is an observation consistent with our previous work in mouse amyloidopathy models [4]. We further demonstrated a positive correlation between tau load and C1q protein levels, and markedly increased C1q staining of tau positive neurons compared to tau negative neurons in P301S mice (Fig. 4a–e), further linking C1q to progression of tau aggregation and hyperphosphorylation in the model [12, 25].

C1q-dependent synapse elimination is firmly implicated in both developmental and pathological synapse loss [2, 5, 26]. Increased C1q at synapses was previously reported in P301S mice [14], while deletion of C1q rescued synapses in this model [2, 14]. In AD models C1q is directly activated by amyloid, decorates amyloid beta plaques in post-mortem AD brains, and genetic knockout of C1q in mouse AD models protects against synapse loss [3]. We explored roles of C1q at the synapse in P301S mice by quantifying C1q-positive synapses in excitatory and inhibitory synaptic subtypes. We found that the loss of excitatory synapses correlated with C1q levels, increased at 6 M when significant synapse loss had occurred (Homer1, Fig. 5c, g). This is consistent with other studies reporting increased C1q tagged synapses in P301S mice at 9M [14], implying that C1q plays an important role in tagging synapses for elimination. In contrast, no such association was seen for inhibitory synapses where the proportion of C1q-positive synapses was consistent across ages despite significant synapse loss at 4–6 M (Gephyrin, Fig. 5k–n). This may be a consequence of the smaller proportion of inhibitory synapses lost (1–1.5 fold decrease compared to WT) compared to excitatory ones (2 fold decrease compared to WT) in the model. Others have reported a significant reduction in gephyrin-positive inhibitory synapses in P301S mice at 9 M in a model utilising the prion promoter to drive overexpression, and showed that elimination of inhibitory synapses is dependent on astrocytes rather than microglia [2]. Our data suggest that excitatory synapses are more vulnerable to complement dysregulation than inhibitory synapses and there may be different mechanisms involved in their elimination (Fig. 5).

To further explore the role of C1q in excitatory synapse loss, the impact of proximity to tau pathology on proportion synapses positive for C1q was tested. Concentric zones were created around neurons containing tau aggregates and healthy neurons, and the proportion of C1q-positive synapses calculated for each zone. The proportion of C1q-labelled synapses around healthy neurons was higher than around tau-positive neurons, likely a consequence of greater synapse loss in the latter case.

Our expression and localisation data implicated complement CP activation as a driver of synapse loss in the tauopathy model. We have recently reported the localisation of the brain-specific CP C3 convertase regulator CSMD1 at the synapse in mouse and man [17]; to understand whether CSMD1 might regulate CP activation at the synapse and restrict synapse loss in P301S mice, synapses were co-labelled with C1q and CSMD1. Although the percentage of C1q positive synapses was decreased in P301S mice compared to WT at 2-4 M, indicative of a lack of complement activation and reflecting the minimal synapse loss at these ages, the percentage of synapses labelled with the CP regulator CSMD1, as well as the percentage of C1q+CSMD1 + synapses, were significantly decreased in P301S mice at 2 and 4 M of age compared to WT, suggesting a heightened vulnerability to complement attack. The proportion of CSMD1 + synapses was small, ~ 15–20%; this may indicate that expression of CSMD1 is limited to specific synaptic subtypes, a subject for further investigation.

To confirm whether the CP dysregulation observed in P301S mice was replicated at the synapse in human tauopathy brain, tissue was obtained from sporadic CBD and GGT tauopathy cases and controls and stained for synapse markers, C1q and CSMD1, essentially as in the mouse. Although no significant differences were detected in the number of excitatory or inhibitory synapses, a non-significant trend towards increased loss of viable excitatory synapses was observed (Fig. 8); consistent with the mouse data, the percentage of CSMD1 synapses and C1q+CSMD1 + synapses was decreased in tauopathy cases, significant for GGT compared to controls (Fig. 8n, p). Post-mortem samples from human tauopathy cases inevitably represent advanced disease stages where complement dysregulation may be a less obvious feature compared to the mouse model where we show that complement dysregulation occurs early in the disease process.

## Conclusion

We demonstrate that synaptic loss in both P301S mice and human tauopathy brain may be attributed to complement classical pathway dysregulation at the synapse, particularly in the proximity of tau pathology. C1q tagging of excitatory synapses and reduced expression of the CP regulator CSMD1 at these synapses may collaborate to exacerbate complement damage and resultant synapse loss. We suggest that anti-complement drugs, already proposed for treatment of AD, may also have a role in therapy of tauopathies [27].

## Supplementary Information

Below is the link to the electronic supplementary material.

Supplementary Material 1

Supplementary Material 2

## Data Availability

Primary data will be available from the corresponding author on reasonable request.
