# Supplementary material for: Dysregulation of complement at the synapse in P301S mice and human tauopathies
Source: Acta Neuropathol Commun. 2026 Jun 10;14:175. doi: 10.1186/s40478-026-02347-2 (PMC13495216; doi:10.1186/s40478-026-02347-2)
Supplement: Supplementary file 1 [file 40478_2026_2347_MOESM1_ESM.docx]

**Online Resource 1: List of primers for qPCR**

| Primer | Sequence (5’🡪3’) |
| --- | --- |
| HPRT_Fwd | AGCGTTGGGCTTACCTCAC |
| HPRT_Rev | GCAAGTCTTTCAGTCCTGTCCA |
| FB_Fwd | CAAGGGAAGAGCCTGACTCG |
| FB_Rev | CCCCGGAATCTCCTTTGCAT |
| C3_Fwd | AGCCATATAAAGAGCCAGCGG |
| C3_Rev | CACTGCCCCATGTTGACCA |
| C2_Fwd | GACAAGGTCAGGTACCGCTG |
| C2_Rev | TCGTAAGAGTAAGGCTGTCGG |
| FI_Fwd | AGCCGTTGTGAAGAAGACCG |
| FI_Rev | TCCGATCACTCGTTTCCTGC |
| Crry_Fwd | CTGCCATTTACTTTGGGTCACT |
| Crry_Rev | CAGTGTTGCAGCGGTAGGTA |
| CR4_Fwd | GATTGATGGCTCGGGTAGCA |
| CR4_Rev | CTGCATCAGGGAGAACCGTG |
| CR3_Fwd | TGTTAACCAGACAGGTGCCC |
| CR3_Rev | CACAGCCTCTGGAGGTACTTG |
| C3aR1_Fwd | AGGATTTGTTGGTGGCTCGC |
| C3aR1_Rev | CTCCATGGCTCAGTCAAGCACA |
| C4b/a_Fwd | CCGAGTGGAGTATGGCTTCA |
| C4b/a_Rev | CTAAGGCCTCACACCTGGC |
| C5_Fwd | GTTTATGCCGCTACCAGCCA |
| C5_Rev | GTGCTGAAATGACGTAGGTTTGT |
| CD59a_Fwd | TGACTCTAAGATTGCAGGGTTG |
| CD59a_Rev | GCAGCACTATCTTGAGCCAC |
| FH_Fwd | ACAGCCAGTATGCAAAGATAATTCC |
| FH_Rev | CATCACTTCCACTTGCCCAAA |
| DAF_Fwd | AAGGAGAGCCTAACACAGGTGG |
| DAF_Rev | CTTAGGTGCCCAGAAACACTGC |
| C5aR1_Fwd | GGCCATCCTGCGGCTGATGG |
| C5aR1_Rev | GCCTTGCGACTCCAGGTCCG |
| C1q_Fwd | TGTCCCACCATCAGCAAAGG |
| C1q_Rev | GTCTCCATGGTGTCCCTGC |
| CSMD1_Fwd | CACTGCAGCTAAGGGTCAGAA |
| CSMD1_Rev | GATGTAGCCGGAGAGACAGC |
